# Preoperative myopenia is associated with postoperative complications and endoscopic recurrence after ileocolic resection in Crohn’s disease

**DOI:** 10.1177/17562848261477206

**Published:** 2026-09-06

**Authors:** Angelo Del Gaudio, Guia Becherucci, Federica Di Vincenzo, Teodoro Pietro Di Certo, Fabio Cascella, Marco Murgiano, Simone Parello, Flavio Fiaccavento, Francesca Bice D’Angelo, Gaetano Coppola, Irene Mignini, Elisa Foscarini, Francesca Profeta, Lucrezia Laterza, Daniele Napolitano, Elisa Schiavoni, Massimo Musumeci, Franco Sacchetti, Paola Caprino, Luigi Sofo, Antonio Gasbarrini, Daniela Pugliese, Alfredo Papa, Franco Scaldaferri, Laura Maria Minordi, Loris Riccardo Lopetuso

**Affiliations:** 1 UOS Malattie Infiammatorie Croniche Intestinali, Centro di Malattie dell’Apparato Digerente (CEMAD), Fondazione Policlinico Universitario “A. Gemelli” IRCCS, 9371 Università Cattolica del Sacro Cuore, Rome, Italy; 2 UOC di Radiologia Addomino-Pelvica, Dipartimento di Diagnostica per Immagini, Radioterapia Oncologica ed Ematologia, Fondazione Policlinico Universitario “A. Gemelli”, IRCCS, 9371 Università Cattolica del Sacro Cuore, Rome, Italy; 3 Dipartimento di Scienze Mediche e Chirurgiche, 9371 Università Cattolica del Sacro Cuore, Rome, Italy; 4 Abdominal Surgery Unit, Department of Gastroenterological Sciences, Fondazione Policlinico Universitario A. Gemelli’ IRCCS, 9371 Università Cattolica del Sacro Cuore, Rome, Italy; 5 UOS Gastroenterologia, Ospedale Isola Tiberina Gemelli Isola, Rome, Italy; 6 Department of Life Science, Health, and Health Professions, Link Campus University, Rome, Italy

**Keywords:** Crohn’s disease, myopenia, endoscopic recurrence, ileocolic resection, postoperative complications

## Abstract

**Background:**

Low muscle mass is increasingly recognised in patients with Crohn’s disease, particularly among those requiring intestinal surgery. While reduced muscle mass has been associated with adverse early surgical outcomes, its relationship with postoperative endoscopic recurrence remains poorly defined.

**Objectives:**

To evaluate the association between preoperative low muscle mass, postoperative complications and endoscopic recurrence after ileocolic resection for Crohn’s disease.

**Design:**

We conducted a retrospective cohort study including consecutive adult patients with Crohn’s disease undergoing ileocolic resection between 2017 and 2024.

**Methods:**

Muscle mass was assessed on preoperative computed tomography or magnetic resonance imaging using the total psoas area index (TPAI) with sex-specific cut-offs. The primary outcome was early postoperative complications (Clavien–Dindo grade ≥II). Secondary outcomes included endoscopic recurrence, defined as Rutgeerts score ≥i2 at follow-up endoscopy (6–12 months after surgery), and clinical factors associated with low muscle mass.

**Results:**

A total of 152 patients were included, of whom 61 (40.1%) were classified as having low muscle mass. Postoperative complications occurred in 25.7% of patients and endoscopic recurrence in 50.7% at 6–12 months. Low muscle mass was independently associated with early postoperative complications (OR 2.80, 95% CI 1.24–6.29) and with endoscopic recurrence (OR 2.20, 95% CI 1.13–4.57). Upper gastrointestinal involvement was independently associated with low muscle mass, whereas prior exposure to biologic therapy showed an inverse association. Body mass index showed only a weak correlation with imaging-based muscle mass.

**Conclusion:**

Preoperative low muscle mass is common in patients with Crohn’s disease undergoing ileocolic resection and was independently associated with both early postoperative complications and endoscopic recurrence. Imaging-based body composition assessment may improve perioperative risk stratification.

## Plain language summary

Crohn’s disease is a long-term inflammatory condition of the digestive tract. Some patients develop loss of muscle mass, particularly when the disease is severe or longstanding. This study investigated whether low muscle mass before surgery is linked to worse outcomes after intestinal surgery for Crohn’s disease. We retrospectively studied 152 patients who underwent ileocolic resection between 2017 and 2024. Muscle mass was measured using routine computed tomography or magnetic resonance imaging scans performed before surgery. About 40% of patients were found to have low muscle mass. These patients were more likely to develop postoperative complications and were also more likely to experience disease recurrence at follow-up endoscopy within the first year after surgery. These associations remained significant after adjustment for other clinical factors. Low muscle mass was more common in patients with upper gastrointestinal involvement. Importantly, many patients with low muscle mass had a normal body weight, suggesting that body mass index alone may fail to identify patients at nutritional risk. Our findings suggest that routine assessment of muscle mass before surgery may help identify patients at higher risk and support more personalized nutritional and postoperative management strategies.

## Graphical abstract

**Graphical Abstract:**
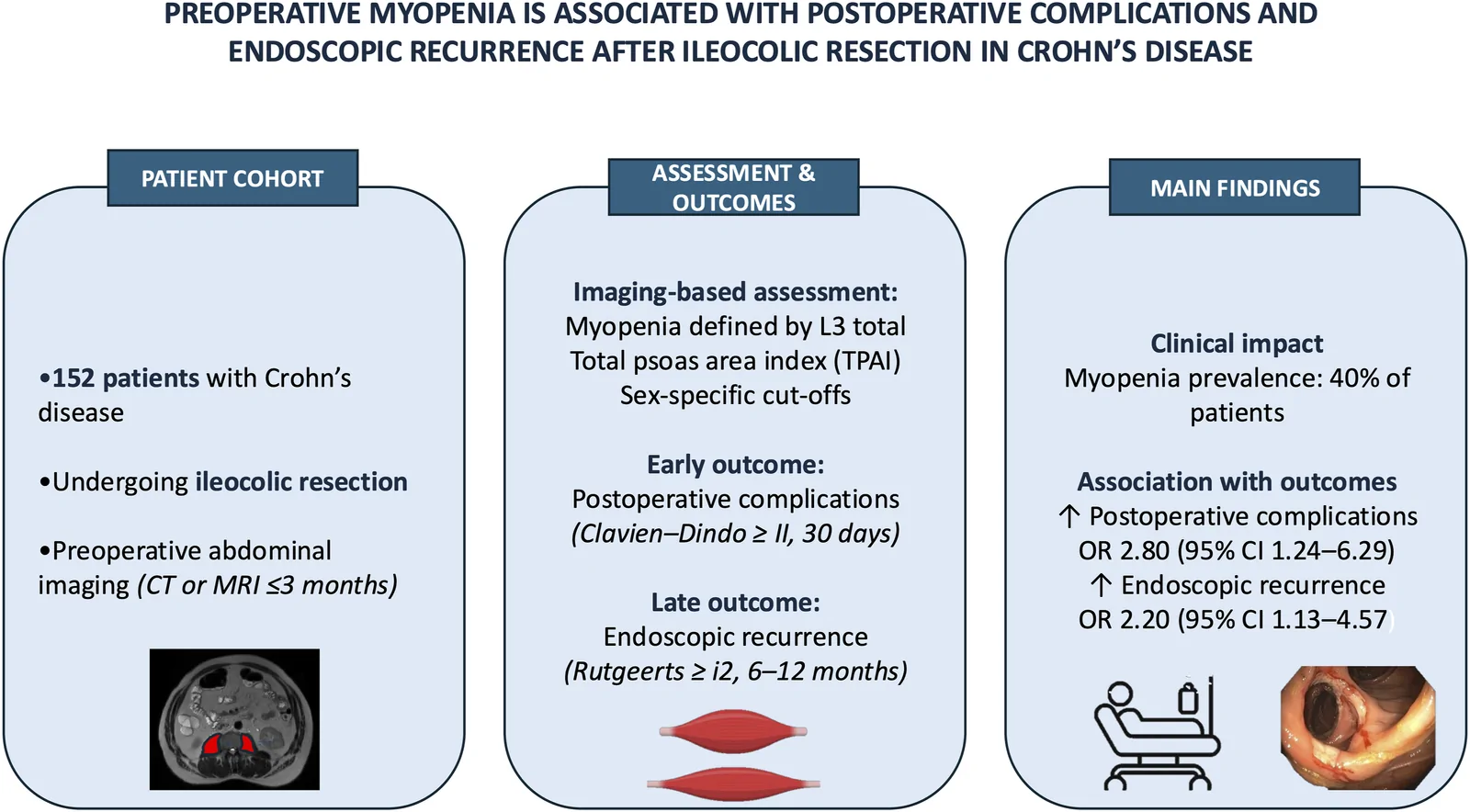

## Introduction

Crohn’s disease (CD) is a chronic inflammatory bowel disease characterized by progressive bowel injury and reduced quality of life.^1^ Chronic inflammation, malnutrition, malabsorption, and increased catabolic activity in CD patients often lead to progressive muscle depletion and impaired body composition.^1^ Sarcopenia is defined as a progressive and generalized skeletal muscle disorder encompassing both muscle mass and function reduction, causing low strength and physical performance.^2^ This condition ranges from 33% to over 60% in patients with CD and appears to be primarily associated with higher Crohn’s disease activity index (CDAI), lower body mass index (BMI), and poorer nutritional status.^3,4^ Although the expanding therapeutic armamentarium for CD has reduced the need for surgery, approximately one quarter of patients still require intestinal resection during the course of disease.^5,6^ Of note, the prevalence of sarcopenia increases up to 61% when considering only CD patients who have undergone surgery, and the presence of sarcopenia has been associated with an increased likelihood of requiring surgery.^7^ Emerging evidence indicates that sarcopenia may predispose patients with CD to adverse postoperative outcomes, including early surgical complications such as anastomotic leak and higher-grade morbidity (Clavien–Dindo grade ≥III) and to early endoscopic postoperative recurrence (ER).^8–10^ Among the imaging-based methods proposed for skeletal muscle mass assessment, measurement of the total psoas area index (TPAI) on CT or MRI scans has emerged as a practical and reproducible surrogate marker.^11,12^ Evidence linking imaging-defined sarcopenia to endoscopic recurrence after ileocolic resection is limited and inconsistent, and the interplay between sarcopenia, early postoperative complications, and subsequent endoscopic recurrence has not been systematically explored. It should be noted that in the existing IBD and surgical literature, the term sarcopenia has frequently been used to describe reduced skeletal muscle mass assessed by cross-sectional imaging alone, without formal evaluation of muscle strength or physical performance. However, according to contemporary consensus definitions, isolated imaging-based reduction in skeletal muscle mass is more accurately referred to as myopenia. Therefore, in the present study, we adopted the term myopenia to specifically indicate imaging-based reduction in skeletal muscle mass. Accordingly, this study aimed to assess the relationship between preoperative myopenia and early postoperative complications and endoscopic recurrence following ileocolic resection in Crohn’s disease, and to identify clinical determinants of myopenia.

## Material and methods

### Study design and patient population

This is a real-life, retrospective, single-center study including consecutive adult patients (≥18 years) with Crohn’s disease (CD) who underwent ileocolic resection (ICR) at the Fondazione Policlinico Universitario A. Gemelli IRCCS between 2017 and 2024. Eligible patients were required to have undergone a preoperative abdominal computed tomography (CT) scan or magnetic resonance imaging (MRI) within 3 months prior to surgery, as well as postoperative follow-up with endoscopic assessment performed approximately 6 to 12 months after surgery or after stoma closure, when applicable, in accordance with routine clinical practice. CT and MRI exams were performed using enterography technique in which an oral contrast media (polyethylene glycol solution, PEG) was used to obtain small bowel distension before IV administration of iodinated contrast medium in CT or paramagnetic contrast medium in MR.

Exclusion criteria included indications for ileocolic resection other than CD, presence of permanent ileostomy, performance of surgical procedures other than ileocolic resection or ileocolic anastomosis resection, and inadequate image quality for the presence of extensive motion or intestinal artifacts on preoperative abdominal imaging. The indication for surgery reflected routine clinical practice at our centre and was generally established through multidisciplinary discussions involving gastroenterologists, surgeons and radiologists. The choice between MRI or CT imaging was based on the referring physician’s clinical judgment, in accordance with institutional protocols and standard clinical practice; both routinely used at our institution for preoperative assessment in our centre. Postoperative biologic prophylactic therapy was prescribed at the discretion of the treating gastroenterologist, based on individual patient characteristics, postoperative risk assessment, and current international guidelines, in accordance with routine clinical practice at our centre. Notably, endoscopic follow-up was performed according to routine clinical practice and not mandated by the study protocol. All consecutive eligible patients meeting inclusion criteria during the study period were included. The reporting of this study conforms to the Strengthening the Reporting of Observational Studies in Epidemiology (STROBE) statement.^13^

### Outcomes

The primary outcome of the study was the occurrence of postoperative complications within 30 days after surgery. Postoperative complications were classified according to the Clavien–Dindo classification (grade ≥II). Secondary outcomes included postoperative endoscopic recurrence (ER), defined as a Rutgeerts score ≥i2, assessed 6–12 months after surgery and the identification of preoperative clinical factors associated with myopenia.^14^

### Data collection and endoscopic assessment

At index surgery (baseline), demographic and clinical data were retrospectively collected from electronic medical records, including age, gender, duration of disease, disease location and behaviour according to Montreal classification, smoking habit, history of perianal disease, BMI, indication for surgery, previous exposure to advanced therapies, prior CD-related intestinal resections, preoperative laboratory parameters. Specifically, preoperative laboratory parameters were collected at the most recent available assessment within 3 months prior to surgery. Preoperative hypoalbuminemia was defined as a serum albumin level <3.5 g/dL, according to the reference values of the institutional laboratory. Postoperative clinical outcomes, including postoperative complications and postoperative medical treatments, were collected during follow-up after surgery.^15^ Postoperative complications were classified by trained and dedicated investigators who were blinded to body composition parameters. Postoperative sepsis was defined as a clinically suspected or confirmed infection requiring intravenous antibiotic therapy, as documented in the medical records. The severity of inflammation at the ileocolic anastomosis and in the neoterminal ileum was assessed using the Rutgeerts score (RS), as derived from endoscopic reports.

### Preoperative body composition analysis

All preoperative abdominal imaging studies (CT or MRI) performed within 3 months prior to surgery were analysed by a dedicated expert radiologist who was blinded to clinical outcomes and endoscopic data, using pseudonymized images. Body composition assessment was performed on a single standardized axial image at the level of the third lumbar vertebra (L3) on both CT and MRI, allowing a standardized evaluation of psoas muscle area across imaging modalities.

Manual freehand region-of-interest measurements were performed using the Carestream picture archiving and communication system (PACS), with muscle areas automatically calculated by the software. In CT examinations, a circular region of interest (ROI) was additionally placed over the psoas muscle at the same L3 level to assess muscle density in Hounsfield units (HU); the calculations were performed in images acquired during portal phase. In MR, the calculations were performed in single-shot T2 weighted sequences. The bilateral psoas muscle areas were manually segmented, and the sum of muscle areas was normalized for patient height, yielding the Total Psoas Area Index (TPAI). Myopenia was assessed both as a continuous variable using the TPAI and as a categorical variable, defining myopenia based on sex-specific TPAI cut-off values; <5.4 cm^2^/m^2^ for men and <3.56 cm^2^/m^2^ for women.^16^

### Statistical analysis

Categorical variables were described as absolute frequencies and percentages, while continuous variables were reported as mean ± standard deviation (SD) or as median and interquartile range (IQR), as appropriate based on data distribution. Differences between groups were assessed using the student’s t test for normally distributed continuous variables, the Mann–Whitney U test for non-normally distributed continuous variables, and the χ^2^ test for categorical variables.

Postoperative complications were classified according to the Clavien–Dindo classification and subsequently dichotomized into complications of grade <II and clinically relevant complications of grade ≥II for statistical analyses. Disease phenotype was categorized as penetrating (B3) versus non-penetrating (B1–B2) according to the Montreal classification. Previous surgical history was considered as a dichotomous variable (patients with at least one prior intestinal surgery versus surgery-naïve patients). Disease duration was analysed as a categorical variable using the median value of the cohort (12 years) as the cutoff, categorizing patients into disease duration ≤12 years and >12 years. Comorbidities were assessed using the Charlson Comorbidity Index and subsequently categorized into two groups (≤2 vs >2) to distinguish patients with low versus high comorbidity burden. Myopenia was evaluated through body composition analysis on preoperative imaging (CT or MRI) and was considered both as a continuous variable, using the Total Psoas Area Index (TPAI) normalized for height, and as a categorical variable, defining myopenia based on sex-specific TPAI cut-off values to account for physiological sex-related differences. The association between preoperative body mass index (BMI), measured at hospital admission for surgery, and body composition parameters was assessed using Spearman’s rank correlation coefficient. Associations between clinical factors and study outcomes (postoperative complications, endoscopic recurrence, and myopenia) were explored using univariate analyses followed by outcome-specific multivariable logistic regression models. Variables included in each multivariable model were selected a priori based on clinical relevance and/or statistical significance at univariate analysis. Candidate covariates included age at surgery, sex, disease duration, comorbidity burden, penetrating disease phenotype, smoking status, pre- and postoperative medical therapy, prior intestinal surgery, hypoalbuminemia, upper gastrointestinal involvement, and myopenia, according to the outcome analysed. The final set of variables differed across models based on the specific outcome investigated. Before inclusion in the final models, multicollinearity among independent variables was assessed to exclude high correlations that could affect the stability of estimates. Model assumptions were checked and goodness-of-fit was assessed. Results of regression analyses were reported as adjusted odds ratios (OR) with corresponding 95% confidence intervals (CI). All OR reported were derived from multivariable logistic regression models. All statistical tests were two-sided, and a p value ≤0.05 was considered statistically significant. Given the retrospective observational design, no formal a priori sample size calculation was performed. All consecutive eligible patients meeting inclusion criteria during the study period were included, reflecting real-world clinical practice at our centre. Multivariable models were constructed parsimoniously according to the number of outcome events in order to minimize the risk of model overfitting. Statistical analyses were performed using Stata software (version 18.5; StataCorp, College Station, TX, USA).

## Results

### Patient characteristics

A total of 177 patients were screened for study inclusion. 10 patients were excluded due to inadequate quality of radiological imaging, 8 patients declined participation, and 7 patients did not continue follow-up at our centre (Supplementary Figure 1). Overall, 152 patients with median age at surgery of 43.9 years were retrospectively included in the study (Table 1). Preoperative imaging consisted of MR enterography in 99 patients and CT enterography in 53 patients. Approximately one-third of patients (28.9%) had undergone previous intestinal resections, and 68 patients (44.7%) were active smokers after surgery. The median disease duration was 12 years. Postoperative biologic prophylactic therapy was administered in 102 (67.1%) of patients. Postoperative complications (Clavien–Dindo ≥II) occurred in 39 patients (25.7%) (Supplementary Table 1). ER (RS ≥i2) at 6–12 months after surgery was observed in 77 patients (50.7%); the distribution of RS is reported in Supplementary Table 2.

**Table 1.** Demographic and clinical features of the study population.

Variable	N (%) or median (IQR)
**Female sex**	71 (46.7%)
**BMI (kg/m** ^**2**^ **)**	21.8 (19.7- 24.2)
**Previous surgeries**	44 (28.9%)
**Age at surgery (years)**	43.9 (31.6 - 54.4)
**Age Montreal Classification:**	
A1 (<17 years)	19 (12.5%)
A2 (17–40 years)	86 (56.5%)
A3 (>40 years)	47 (30.9%)
**Behavior Montreal Classification**	
Penetrating disease (B3)	53 (34.8%)
**Upper GI involvement**	21 (13.8%)
**Active Smoking**	68 (44.7%)
**Disease duration (≥12 years)**	79 (51.9%)
**Preoperative biologic therapy**	84 (55.2%)
**Postoperative biologic prophylactic therapy**	102 (67.1%)
**Postoperative complications (Clavien-Dindo ≥II)**	39 (25.7%)
**Endoscopic recurrence**	77 (50.7%)
**Positive surgical margins**	50 (32.8%)
**Comorbidities (Charlson ≥2)**	49 (32.2%)
**TPAI (cm** ^**2**^ **/m** ^**2**^ **)**	4.6 (3.5 – 6.2)
**Hypoalbuminemia**	70 (46.0%)
**Myopenia (sex specific TPAI cut-offs)**	61 (40.1%)

Continuous variables are expressed as median and interquartile range(IQR), and categorical variables as n (%). Abbreviations: BMI, body mass index; GI, gastrointestinal; TPAI, total psoas area index.

### Preoperative myopenia and associated clinical characteristics

**Figure 1.**
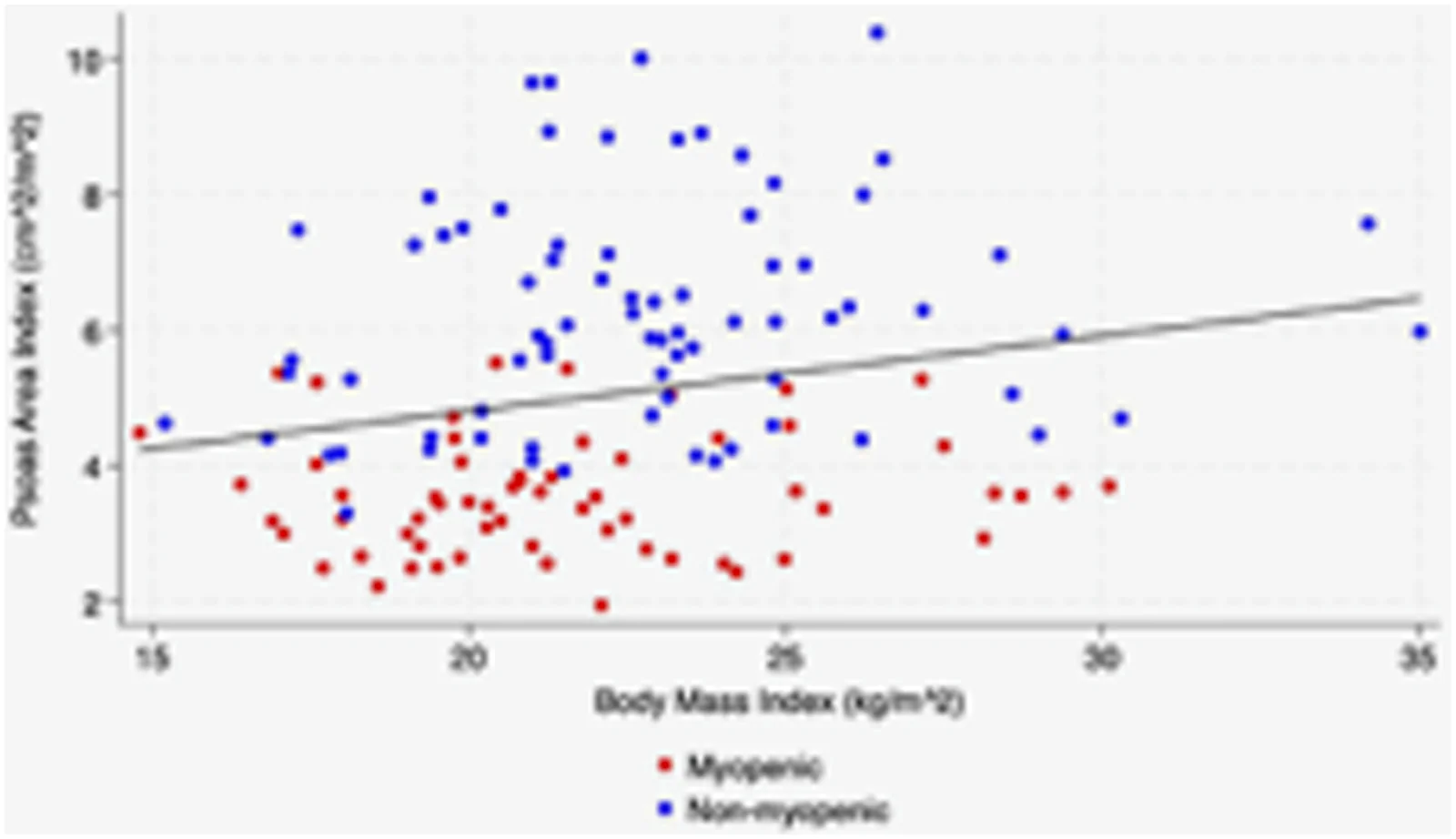
Association between body mass index and total psoas area index Scatter plot showing the association between body mass index (BMI) and total psoas area index (TPAI) in patients with Crohn’s disease undergoing ileocolic resection. Each point represents an individual patient; red dots indicate myopenic patients and blue dots non-myopenic patients, as defined by sex-specific TPAI cut-offs. A weak positive association between BMI and TPAI was observed, with substantial overlap between groups across the entire BMI spectrum. The line represents a fitted linear trend for visualization purposes. The association was evaluated using Spearman’s rank correlation coefficient (ρ).

Median TPAI values in the overall cohort were 4.6 cm^2^/m^2^, using sex-specific cutoff values; 61 patients (40.1%) were classified as having myopenia. Myopenic patients had a significantly lower BMI compared with non-myopenic patients (20.8 vs 22.7 kg/m^2^, p = 0.017) (Table 2). Among patients classified as myopenic, only 16.4% had a BMI <18.5 kg/m^2^, whereas 83.6% had a BMI within the non-underweight range according to conventional BMI-based criteria. Correlation between BMI and TPAI observed in the overall cohort showed a weak but significant positive correlation (Spearman’s ρ = 0.26, p = 0.002) (Figure 1). Myopenic patients showed a longer disease duration (≥12 years: 62.3% vs 45.1%, p = 0.037), higher prevalence of history of upper gastrointestinal (GI) involvement (22.9% vs 7.7%, p = 0.008), higher prevalence of preoperative hypoalbuminemia (57.3% vs 38.5%, p=0.022). On multivariable logistic regression analysis, history of upper GI involvement (OR 5.19, 95% CI 1.63 – 16.55, p = 0.005), and hypoalbuminemia (OR 2.88, 95% CI 1.29–6.44, p = 0.010) were independently associated with an increased likelihood of myopenia. Conversely, preoperative exposure to biologic therapy (OR 0.54, 95% CI 0.17–0.92, p = 0.032) and higher BMI (OR 0.84, 95% CI 0.74–0.96, p = 0.012) were independently associated with a lower risk of myopenia. Longer disease duration showed a trend toward association with myopenia (OR 2.03, 95% CI 0.92–4.46, p = 0.076) (Table 3).

**Table 2.** Univariable comparison of clinical characteristics between myopenic and non-myopenic patients.

Variable	Myopenic N(%) or median (IQR)	Non-myopenic N(%) median (IQR)	p-value
**Postoperative complications (Clavien-Dindo≥ II)**	24 (39.3%)	15 (16.4%)	0.002
**Endoscopic recurrence**	38 (62.3%)	39 (42.8%)	0.019
**BMI (kg/m** ^**2**^ **)**	20.8 (19.2 - 23.2)	22.7 (20.8 - 24.8)	0.017
**Positive surgical margins**	22 (36.0%)	28 (30.7%)	0.481
**Previous surgeries**	20 (32.7%)	24 (26.3%)	0.393
**Postoperative biologic prophylactic therapy**	36 (59.0%)	66 (72.5%)	0.082
**Age Montreal classification**			0.659
A1 (<17 years)	9 (14.7%)	10 (10.9%)	
A2 (17–40 years)	32 (52.4%)	54 (59.3%)	
A3 (>40 years)	20 (32.7%)	27 (29.6%)	
**Behavior Montreal Classification**			0.138
Penetrating disease (B3)	17 (27.8%)	36 (39.5%)	​
**Active Smoking**	23 (37.7%)	45 (49.4%)	0.179
**Age at surgery (years)**	43.9 (30.4- 54.4)	42.7 (31.7 - 52.8)	0.704
**Comorbidities (Charlson ≥2)**	24 (39.3%)	25 (27.4%)	0.219
**Disease duration (≥12 years)**	38 (62.3%)	41 (45.0%)	0.037
**Upper GI involvement**	14 (22.9%)	7 (7.6%)	0.008
**Hypoalbuminemia**	35 (57.3%)	35 (38.4%)	0.022
**Preoperative biologic therapy**	31 (50.8%)	53 (58.2%)	0.367

Continuous variables are expressed as median and interquartile range(IQR), and categorical variables as n (%). Abbreviations: BMI, body mass index; GI, gastrointestinal; TPAI, total psoas area index.

**Table 3.** Multivariable logistic regression analysis of factors associated with myopenia.

Variable	OR	95% CI	p-value
Upper GI involvement	5.19	1.63 – 16.55	0.005
Disease duration (≥12 years)	2.03	0.92 – 4.46	0.076
Preoperative biologic therapy	0.54	0.17 – 0.92	0.032
Penetrating disease (B3)	0.54	0.23 – 1.25	0.155
Hypoalbuminemia	2.88	1.29 – 6.44	0.010
Age at surgery (years)	1.01	0.99 – 1.04	0.203
BMI (kg/m^2^)	0.84	0.74 – 0.96	0.012

Odds ratios (OR) with 95% confidence intervals (CI) and p-values are shown.

### Post-operative early complications

**Figure 2.**
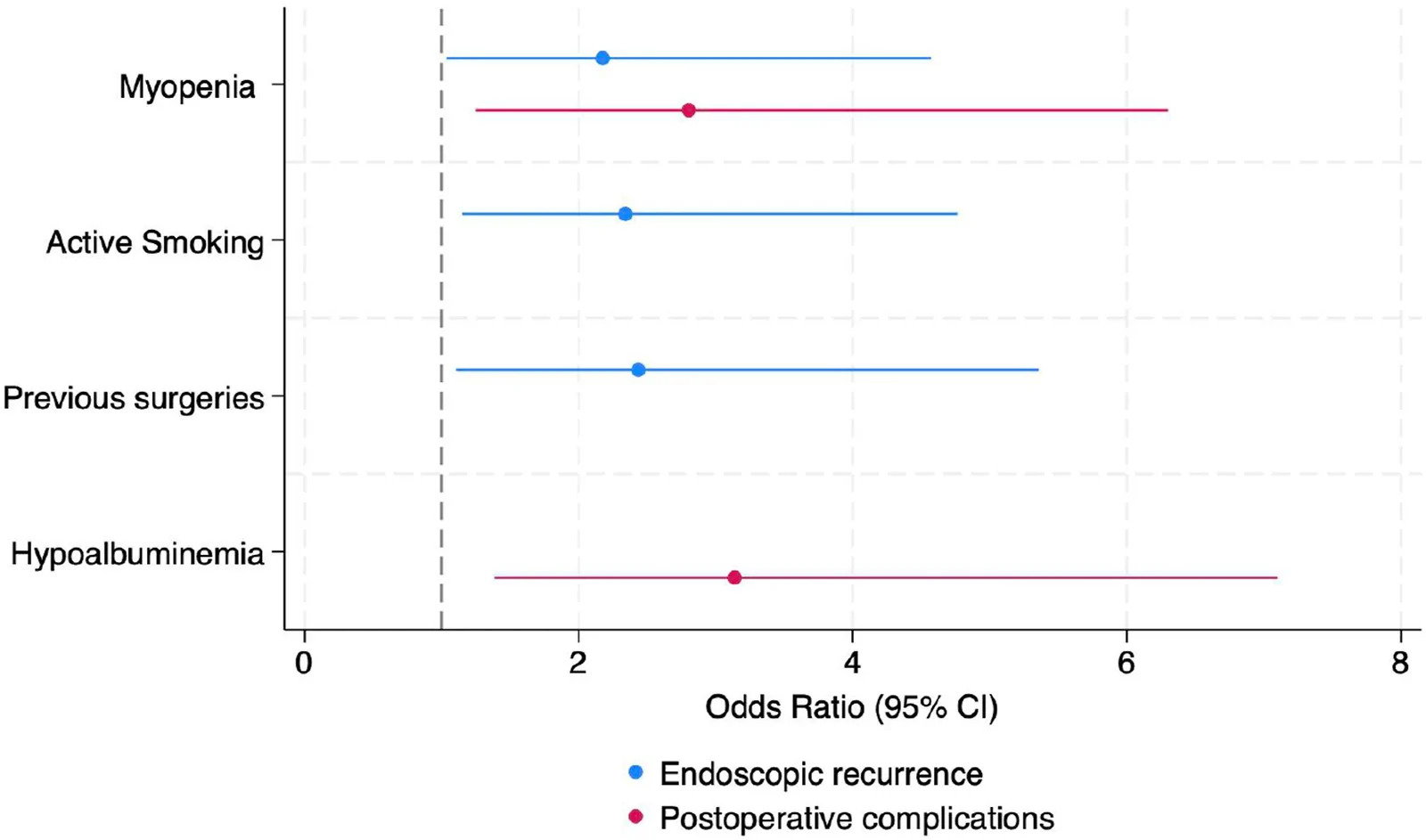
Forest plot of multivariable predictors of postoperative outcomes Forest plot showing independent predictors of endoscopic recurrence and postoperative complications. Odds ratios (ORs) with 95% confidence intervals (CIs) were derived from multivariable logistic regression models. Blue markers represent predictors of endoscopic recurrence, while red markers represent predictors of postoperative complications. Myopenia was independently associated with both outcomes. The vertical dashed line indicates an OR of 1.0.

Septic complications were the most frequent, occurring in 27 patients (17.8%). Eight patients (5.5%) required reoperation within 30 days after surgery. Patients who developed postoperative complications had a significantly higher prevalence of myopenia (61.5% vs 32.7, p = 0.002), as well as a higher prevalence of preoperative hypoalbuminemia (69.2% vs 38.0 %, p = 0.006) (Table 4). On multivariable logistic regression analysis, both myopenia (OR 2.80, 95% CI 1.24 – 6.29, p = 0.002) and preoperative hypoalbuminemia (OR 3.13, 95% CI 1.38 – 7.10, p = 0.006) were confirmed as independent predictors of postoperative complications (Table 5) (Figure 2). A sensitivity analysis was performed using a supplementary multivariable model including BMI instead of myopenia. In this model, BMI was not independently associated with postoperative complications, while hypoalbuminemia remained significantly associated with the outcome (OR 2.65, 95% CI 1.12–6.23, p = 0.025) (Supplementary Table 3).

**Table 4.** Univariable comparison between patients with and without postoperative complications.

Variable	Complications N(%) or median (IQR)	No complications	p-value
		N(%) median (IQR)	
**Postoperative biologic prophylactic therapy**	26 (66.6%)	76 (67.2%)	0.946
**Endoscopic recurrence**	24 (61.5%)	53 (46.9%)	0.115
**Myopenia**	24 (61.5%)	37 (32.7%)	0.002
**BMI (kg/m** ^**2**^ **)**	21.2 (19.1 - 23.8)	22.1 (20.0 - 24.3)	0.244
**Positive surgical margins**	15 (38.4%)	35 (30.9%)	0.384
**Montreal Classification**			0.856
A1 (<17 years)	4 (10.2%)	15 (13.2%)	
A2 (17–40 years)	22 (56.4%)	64 (56.6%)	-
A3 (>40 years)	13 (33.3%)	34 (30.0%)	-
**Behavior Montreal Classification**			0.311
Penetrating disease (B3)	11 (28.2%)	42 (37.1%)	
**Active Smoking**	15 (38.4%)	53 (46.9%)	0.338
**Female sex**	20 (51.2%)	51 (45.1%)	0.567
**Age at surgery (years)**	48.0 (34.0 - 57.6)	41.5 (30.0 - 53.9)	0.177
**Previous surgeries**	13 (33.3%)	31 (27.4%)	0.331
**Preoperative biologic therapy**	20 (51.2%)	64 (56.6%)	0.562
**Hypoalbuminemia**	27 (69.2%)	43 (38.0%)	0.001
**Comorbidities (Charlson ≥2)**	14 (35.9%)	35 (30.9%)	
**Disease duration (≥12 years)**	24 (61.5%)	55 (48.6%)	0.166
**Upper GI involvement**	5 (12.8%)	16 (14.1%)	0.835

Continuous variables are expressed as median and interquartile range(IQR), and categorical variables as n (%). Abbreviations: BMI, body mass index; GI, gastrointestinal.

**Table 5.** Multivariable logistic regression predictors of postoperative complications.

Variable	OR	95% CI	p-value
Disease duration (≥12 years)	1.28	0.57 – 2.88	0.539
Hypoalbuminemia	3.13	1.38 – 7.10	0.006
Comorbidities	0.69	0.27 – 1.75	0.439
Age at surgery (years)	1.02	0.99 – 1.05	0.162
Myopenia	2.80	1.24 – 6.29	0.002
Active Smoking	0.74	0.33 – 1.66	0.797

Odds ratios (OR) with 95% confidence intervals (CI) and p-values are shown.

### Post operative endoscopic recurrence

Patients with ER had a significantly higher prevalence of prior resection (36.3% vs 21.3%, p = 0.041). and myopenia compared with patients in endoscopic remission (49.3% vs 30.7%, p = 0.019) (Table 6). A trend toward a higher recurrence rate was observed in active smokers (51.9% vs 37.3%, p = 0.059) and in patients with older age at surgery (p = 0.052). No significant differences were observed between groups in terms of postoperative medical therapy, postoperative complications, BMI, surgical margin status, or penetrating disease behavior (p = 0.773). On multivariable logistic regression analysis, myopenia (OR 2.20, 95% CI 1.13 – 4.57, p = 0.040), active smoking (OR 2.13, 95% CI 1.15–4.76, p = 0.019), and prior surgical procedures (OR 2.23, 95% CI 1.10–5.35, p = 0.027) were confirmed as independent predictors of ER (Table 7, Figure 2).

**Table 6.** Univariable Comparison Between Patients With and Without endoscopic recurrence.

Variable	Endoscopic recurrence n(%)	Endoscopic remission n(%)	p-value
**Postoperative biologic prophylactic therapy**	52 (67.5%)	50 (66.6%)	0.910
**Postoperative complications (Clavien-Dindo≥II)**	24 (31.1%)	15 (20.0%)	0.115
**Myopenia**	38 (49.3%)	23 (30.6%)	0.019
**BMI (kg/m** ^**2**^ **)**	21.2 (19.5 - 23.4)	22.2 (19.9 - 24.8)	0.122
**Positive surgical margins**	29 (37.6%)	21 (28.0%)	0.205
**Age Montreal Classification**			0.710
A1 (<17 years)	8 (10.3%)	11 (14.6%)	
A2 (17–40 years)	44 (57.1%)	42 (56.0%)	
A3 (>40 years)	25 (32.4%)	22 (29.3%)	
**Behavior Montreal Classification**			0.773
Penetrating disease (B3)	26 (33.7%)	27 (36.0%)	
**Active Smoking**	40 (51.9%)	28 (37.3%)	0.059
**Female sex**	37 (50.6%)	32 (42.6%)	0.413
**Age at surgery (years)**	45.8 (34.0 - 57.3)	39.6 (27.7 - 52.5)	0.052
**Previous surgeries**	28 (36.3%)	16 (21.3%)	0.041
**Disease duration (≥12 years)**	39 (50.6%)	40 (53.3%)	0.741
**Upper GI involvement**	11 (14.2%)	10 (13.3%)	0.865

Continuous variables are expressed as median and interquartile range(IQR), and categorical variables as n (%). Abbreviations: BMI, body mass index; GI, gastrointestinal.

**Table 7.** Multivariable logistic regression predictors of endoscopic recurrence.

Variable	OR	95% CI	p-value
Age at surgery (years)	1.02	0.995 – 1.044	0.101
Myopenia	2.20	1.13 – 4.57	0.040
Postoperative complications	1.26	0.64 – 3.38	0.358
Postoperative biologic prophylactic therapy	1.39	0.69 – 3.11	0.311
Penetrating disease (B3)	1.21	0.40 – 1.77	0.664
Active Smoking	2.13	1.15 – 4.76	0.019
Previous surgeries	2.23	1.10 – 5.35	0.027

Odds ratios (OR) with 95% confidence intervals (CI) and p-values are shown.

## Discussion

Low muscle mass is a frequent finding in CD, with prevalence rates up to 60% in surgical cohorts and wide variability across studies, reflecting differences in disease activity, nutritional status, patient selection, and assessment methods.^3,4^ In our cohort, approximately 40% of patients met imaging-based criteria for myopenia. In this study, history of upper GI involvement was independently associated with myopenia. Upper GI disease is known to cause pronounced nutritional compromise through impaired oral intake and malabsorption, all of which may accelerate muscle catabolism.^17^ Consistently, proximal small bowel involvement has been associated with worse nutritional status.^18^ We also observed a trend toward an association between longer disease duration and myopenia, with patients affected for ≥12 years demonstrating a tendency toward higher risk, although statistical significance was not reached. This finding is biologically plausible and consistent with prior studies suggesting cumulative muscle loss in IBD driven by chronic systemic inflammation, recurrent disease flares, and persistent nutritional impairment.^19^

Conversely, prior exposure to biologic therapy was inversely associated with the presence of myopenia; however, this finding should be interpreted with caution given the observational design of the study and the relatively wide confidence interval surrounding the estimate. Nevertheless, although speculative, this observation is consistent with emerging evidence suggesting that effective control of systemic inflammation through biologic therapy may contribute to preserving muscle mass by reducing catabolic burden and improving overall nutritional status.^19–21^ A growing body of evidence from cohort studies and meta-analyses underscores the detrimental impact of reduced muscle mass on postoperative outcomes in CD.^3,7,22^ In line with these data, our study demonstrated that myopenia is an independent predictor of postoperative complications in CD patients as graded by the Clavien–Dindo classification. Reduced muscle mass likely reflects diminished physiological reserve and impaired response to surgical stress, contributing to increased susceptibility to complications.^21,22^ Beyond short-term surgical outcomes, an important finding of this study is the independent association between preoperative myopenia and ER. Notably, this association persisted even after adjustment for postoperative complications, suggesting that myopenia may identify a distinct vulnerability phenotype influencing postoperative disease course. Data addressing the relationship between body composition and postoperative recurrence in CD remain limited, and this finding adds novel evidence linking muscle depletion to unfavourable postoperative disease course. ER represents an early and objective marker of ongoing disease activity and is a strong predictor of subsequent clinical relapse and progressive bowel damage, and the association with myopenia may reflect shared underlying mechanisms, including chronic systemic inflammation, and immune dysregulation.^23^ In this context, myopenia may identify patients at higher risk of an unfavourable postoperative disease course, beyond traditional clinical risk factors.^24^ Notably, the observation that the majority of myopenic patients were not underweight according to conventional BMI criteria highlights the limitations of BMI as a surrogate marker of nutritional status. Indeed, BMI does not distinguish between lean and fat mass and may therefore fail to identify clinically relevant muscle depletion and myopenia.^25^ These findings should nevertheless be interpreted in light of several study limitations, including its retrospective single-centre design and the lack of direct assessment of muscle strength or physical performance, which are components of contemporary sarcopenia definitions. Although corticosteroid exposure may represent a relevant confounding factor affecting muscle mass and clinical outcomes, detailed information regarding treatment duration, cumulative exposure, and number of treatment courses was not consistently available across the cohort and could therefore not be included in the analysis. Moreover, data regarding perioperative nutritional interventions, including enteral nutrition support, were not systematically available and could therefore not be accounted for, although such factors may influence both nutritional status and postoperative outcomes. Similarly, standardized preoperative assessment of disease activity was not available; however, all patients included in the study were referred for ileocolic resection due to CD, suggesting the presence of clinically significant disease burden across the study population. Furthermore, data regarding surgical indication (elective versus urgent surgery) were not systematically available and therefore could not be accounted for in the analysis, representing a potential source of residual confounding when evaluating postoperative outcomes. In addition, body composition was assessed using preoperative imaging obtained with different modalities (CT or MRI). Nonetheless, relying on routinely available preoperative imaging and clinically meaningful outcomes enhances the real-world applicability of our findings.

## Conclusion

In our cohort, preoperative myopenia was common among patients with Crohn’s disease undergoing ileocolic resection and was independently associated with both early postoperative complications and endoscopic recurrence. These findings support the potential role of preoperative body composition assessment in perioperative risk stratification.

## Supplemental material

Supplemental material - Preoperative myopenia is associated with postoperative complications and endoscopic recurrence after ileocolic resection in Crohn’s disease Supplemental material for Preoperative myopenia is associated with postoperative complications and endoscopic recurrence after ileocolic resection in Crohn’s disease by Angelo Del Gaudio, Guia Becherucci, Federica Di Vincenzo, Teodoro Pietro Di Certo, Fabio Cascella, Marco Murgiano, Simone Parello, Flavio Fiaccavento, Francesca Bice D’Angelo, Gaetano Coppola, Irene Mignini, Elisa Foscarini, Francesca Profeta, Lucrezia Laterza, Daniele Napolitano, Elisa Schiavoni, Massimo Musumeci, Franco Sacchetti, Paola Caprino, Luigi Sofo, Antonio Gasbarrini, Daniela Pugliese, Alfredo Papa, Franco Scaldaferri, Laura Maria Minordi, Loris Riccardo Lopetuso MD PhD in Therapeutic Advances in Gastroenterology

Supplemental material - Preoperative myopenia is associated with postoperative complications and endoscopic recurrence after ileocolic resection in Crohn’s disease Supplemental material for Preoperative myopenia is associated with postoperative complications and endoscopic recurrence after ileocolic resection in Crohn’s disease by Angelo Del Gaudio, Guia Becherucci, Federica Di Vincenzo, Teodoro Pietro Di Certo, Fabio Cascella, Marco Murgiano, Simone Parello, Flavio Fiaccavento, Francesca Bice D’Angelo, Gaetano Coppola, Irene Mignini, Elisa Foscarini, Francesca Profeta, Lucrezia Laterza, Daniele Napolitano, Elisa Schiavoni, Massimo Musumeci, Franco Sacchetti, Paola Caprino, Luigi Sofo, Antonio Gasbarrini, Daniela Pugliese, Alfredo Papa, Franco Scaldaferri, Laura Maria Minordi, Loris Riccardo Lopetuso MD PhD in Therapeutic Advances in Gastroenterology

## Data Availability

Individual participant data will not be shared. Data are available upon reasonable request. Qualified researchers should contact our team of study coordinators for non-profit studies.*
