# Supplementary material for: Preoperative myopenia is associated with postoperative complications and endoscopic recurrence after ileocolic resection in Crohn’s disease
Source: Ther Adv Gastroenterol. 2026 Sep 6;19:17562848261477206. doi: 10.1177/17562848261477206 (PMC13554609; doi:10.1177/17562848261477206)
Supplement: Supplementary file 1 [file sj-pdf-1-tag-10.1177_17562848261477206.pdf]

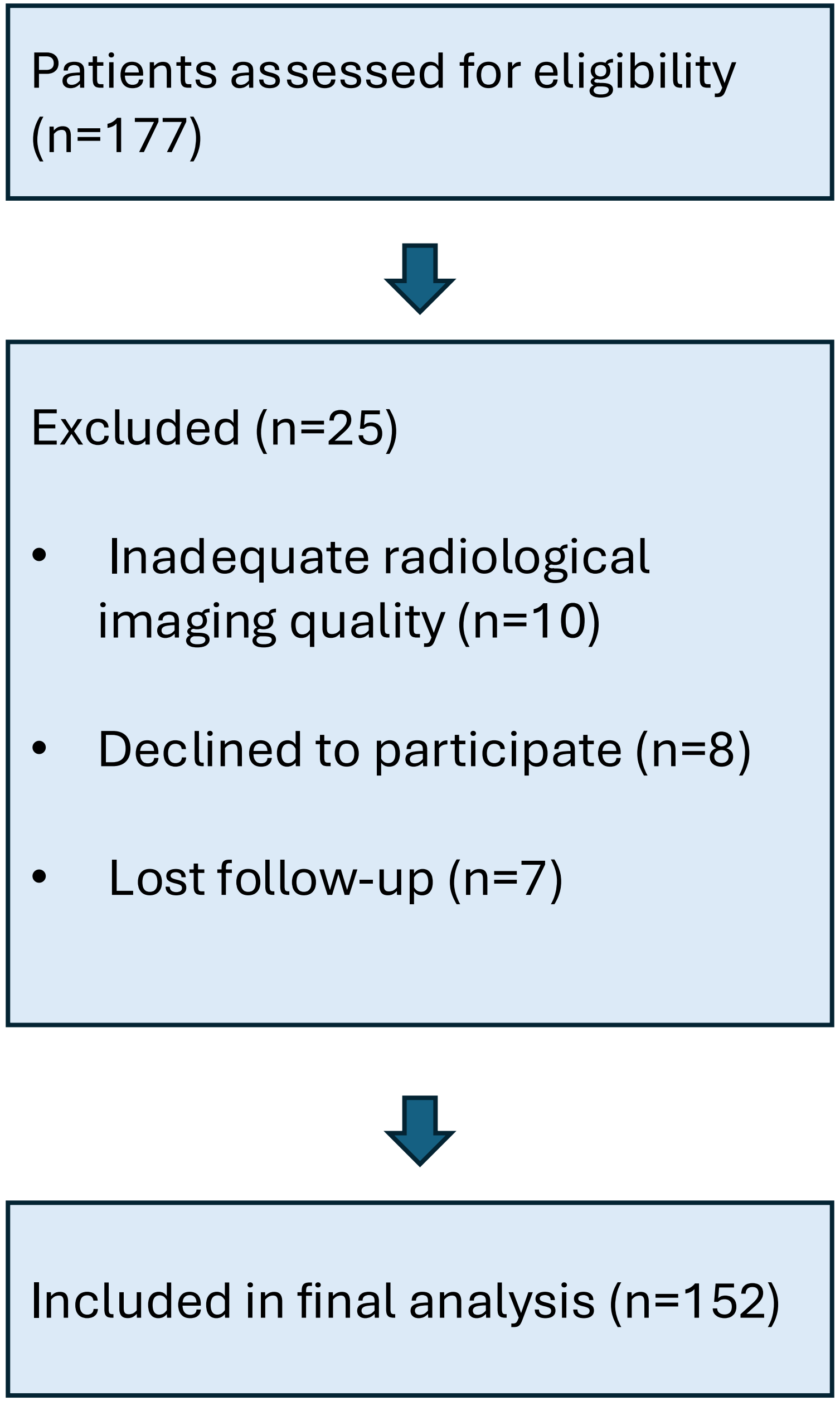
Patients assessed for eligibility (n=177)
Excluded (n=25)
• Inadequate radiological imaging quality (n=10)
• Declined to participate (n=8)
• Lost follow-up (n=7)
Included in final analysis (n=152)
