# Supplementary material for: Preoperative myopenia is associated with postoperative complications and endoscopic recurrence after ileocolic resection in Crohn’s disease
Source: Ther Adv Gastroenterol. 2026 Sep 6;19:17562848261477206. doi: 10.1177/17562848261477206 (PMC13554609; doi:10.1177/17562848261477206)
Supplement: Supplementary file 2 [file sj-pdf-2-tag-10.1177_17562848261477206.pdf]

Supplementary Table 1 postoperative complication

**Variable**	n	%
Postoperative complications (Clavien-Dindo>=2	39	25.7
Postoperative complications (Clavien-Dindo>=3	8	5.3
Anastomotic leak	8	5.5
Anastomotic dehiscence	5	3.3
Reoperation	8	5.3
Preoperative intra-abdominal absces	9	5.9
Postoperative sepsis	27	17.8
Bleeding	8	5.3
Wound infection	9	5.9

Data are reported as number of patients and percentage. Individual patients may have experienced more than one postoperative complication; therefore, percentages do not sum to 100%. Clavien–Dindo grades are reported cumulatively

Supplementary Table 2- Rutgeerts score applied at 6-12 months after surgery

**RUTGEERTS SCORE (n)**
**i0 (56)**
**i1 (19)**
**i2 (39)**
**i3 (22)**
**i4 (16)**
**Total (152)**

Supplementary Table 3. Sensitivity analysis: multivariable logistic regression model for predictors of postoperative complications including BMI

Variable	OR	95% CI	p-value
**Disease duration (≥12 years)**	1.46	0.63 – 3.40	0.371
**Hypoalbuminemia**	2.65	1.12 – 6.23	0.025
**Comorbidities**	1.36	0.50 – 3.70	0.536
**Age at surgery (years)**	1.00	0.97 – 1.04	0.622
**BMI (kg/m²)**	0.91	0.80 – 1.05	0.215
**Active Smoking**	0.83	0.36 – 1.91	0.666

Odds ratios (OR) with 95% confidence intervals (CI) and p-values are shown.
